# The complete mitochondrial genome of *Leucoptera coffeella* (Lepidoptera: Lyonetiidae) and phylogenetic relationships within the Yponomeutoidea superfamily

**DOI:** 10.1038/s41598-024-57869-3

**Published:** 2024-03-26

**Authors:** Mateus Pereira dos Santos, Ana Paula Zotta Mota, Roberto Coiti Togawa, Natalia Florencio Martins, Eliza Fabricio de Melo Bellard do Nascimento, Vivian Santos Lucena, Maria Aparecida Castellani, Erika Valéria Saliba Albuquerque, Frédérique Hilliou

**Affiliations:** 1 Department of Crop Science and Animal Science, State University of Southwestern Bahia, Vitória da Conquista, 45083-300 Brazil; 2 INRAE, Institut Sophia Agrobiotech, Université Côte D’Azur, CNRS, Sophia Antipolis, France; 3 Embrapa Genetic Resources & Biotechnology, Brasília, 70770-917 Brazil; 4 EMBRAPA Agroindústrial Tropical, Fortaleza, CE 60511-110 Brazil

**Keywords:** Entomology, Phylogeny

## Abstract

The coffee leaf miner (*Leucoptera coffeella*) is one of the major pests of coffee crops in the neotropical regions, and causes major economic losses. Few molecular data are available to identify this pest and advances in the knowledge of the genome of *L. coffeella* will contribute to improving pest identification and also clarify taxonomy of this microlepidoptera. *L. coffeella* DNA was extracted and sequenced using PacBio HiFi technology. Here we report the complete *L. coffeella* circular mitochondrial genome (16,407 bp) assembled using Aladin software. We found a total of 37 genes, including 13 protein-coding genes (PCGs), 22 transfer RNA genes (tRNAs), 2 ribosomal RNA genes (rRNAs) and an A + T rich-region and a D-loop. The *L. coffeella* mitochondrial gene organization is highly conserved with similarities to lepidopteran mitochondrial gene rearrangements (trnM-trnI-trnQ). We concatenated the 13 PCG to construct a phylogenetic tree and inferred the relationship between *L. coffeella* and other lepidopteran species. *L. coffeella* is found in the Lyonetiidae clade together with *L. malifoliella* and *Lyonetia clerkella*, both leaf miners. Interestingly, this clade is assigned in the Yponomeutoidea superfamily together with Gracillariidae, and both superfamilies displayed species with leaf-mining feeding habits.

## Introduction

*Leucoptera coffeella* (Guèrin-Meneville & Perrotet 1842) (Lepidoptera: Lyonetiidae) is a monophagous pest in coffee crops of Neotropical America where it causes important economic losses^1^. In Brazil, the world’s largest coffee producer, the negative impact corresponds to more than 50% of the production costs. However, in cases of severe infestations, the damage can compromise up to 70% of the costs^2,3^.

The coffee leaf miner is a microlepidoptera that consumes the palisade parenchyma during the larval stages. The mines reduce the photosynthetic area and induce premature leaf senescence, leading to leaf abscission and, consequently, decreasing the coffee grain yields^4,5^. Despite of the extensive occurrence of this pest and its agronomic importance in coffee growing areas, only a few DNA markers are currently available to monitor the presence of *L. coffeella* in coffee plantations and to characterize its phylogeographic and phylogenetic origins^6^.

Mitochondrial genomes (mitogenomes) are extensively used in differentiation studies to infer phylogenetic relationships^7^ and to develop species-specific molecular markers. However, data from families with large body size species are more abundant than small species, and species-rich tropical ecosystems are usually poorly investigated compared to temperate region faunas^8^. Among the 109 families in Lepidoptera, mitogenomes of Yponomeutoidea superfamily and Lyonetiidae family are poorly represented^9,10^. There are 11 families in Yponomeutoidea (Argyresthiidae, Attevidae, Bedelliidae, Glyphipterigidae, Heliodinidae, Lyonetiidae, Plutellidae, Praydidae, Scythropiidae, Yponomeutidae, Ypsolophidae) with 269 out of the 452 species found in NCBI classified in these families. Only nine families of Yponomeutoidea have complete mitochondrial genome data.

The mitochondrial DNA (mtDNA) has a higher mutation rate than the nuclear one and, as a result of its high copy number, large amounts of mtDNA can be assembled from genomic sequencing^11–14^. Bioinformatics analysis of the mitogenome features allows the determination of its gene size and arrangement, base composition, codon usage, and tRNA secondary structure, which are used to classify insects taxonomically and to assess their evolutionary history.

Insect mitogenomes are relatively small, with highly conserved structures, rapid evolution rates, low levels of recombination, and maternal inheritance. These 14-19kb length double-stranded circular molecules encode 13 Protein-Coding Genes (PCGs): two ATPase (ATP6 and ATP8), three cytochrome c oxidase genes (COI, COII, COIII), one cytochrome b (CYTB), seven NADH dehydrogenase genes (NADH1-6 and NADH4L), 22 transfer RNAs (tRNA), two ribosomal RNA genes (rrnS and rrnL) and a non-coding A + T-rich region^9,11, 15–20^.

In order to fulfill the knowledge gap of mitogenome data of the coffee leaf miner and other leaf-mining insects from the Yponomeutoidea group, we assembled and analyzed the *L. coffeella* mitogenome for sequencing data recently obtained by Martins et al.^21^.

## Results and discussion

### Mitochondrial genome organization and base composition

**Figure 1:**
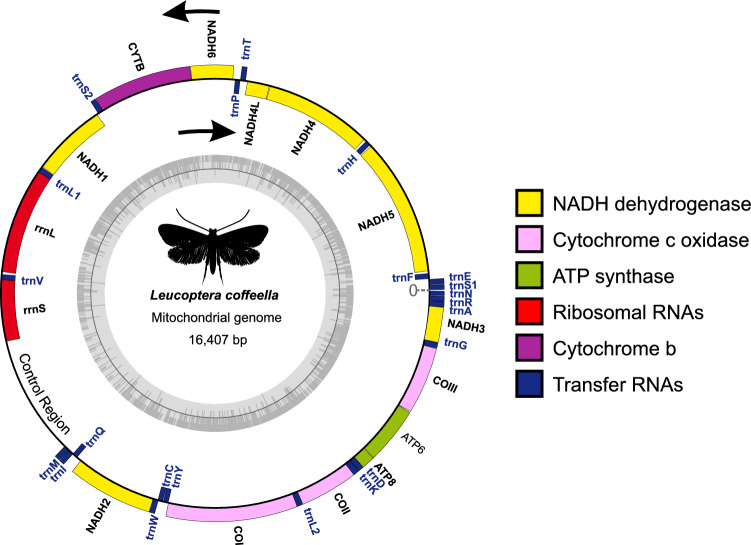
The circular mitochondrial genome of *Leucoptera coffeella*. The J-strand (+) is visualized on the outer circle, and the N-strand (−) on the inner circle.

We assembled the complete mitochondrial genome of *L. coffeella* which consisted in a 16,407 bp circular DNA. Our genome contains 13 PCGs, two rRNA, 22 tRNA genes, and an A + T-rich region. Four of the 13 PCGs (NADH1, NADH4L, NADH4, and NADH5), 8 tRNAs (trnY, trnC, trnQ, trnV, trnL1, trnP, trnH and trnF) and the two rRNAs (rrnS and rrnL) are encoded by the minority-strand while the remaining 23 genes are encoded by the majority strand (Fig. 1 and Table 1). This strand specific genes organization of *L. coffeella* mitogenome is highly conserved with either the evolutionary closed *L. malifoliella* or the more distant Bombycoidea insects^10,22^.

**Table 1:** Annotation of the *Leucoptera coffeella* mitochondrial genome.

Gene (anticodon)	Start	Stop	Strand	Size	Intergenic nucleotides	Start/stop codon
trnS1(gct)	42	102	+	61	4	
trnE(ttc)	107	172	+	66	5	
trnF(gaa)	178	245	−	68	27	
NADH5	273	1985	−	1713	15	ATT/TAA
trnH(gtg)	2001	2068	−	68	28	
NADH4	2097	3437	−	1341	7	ATG/TAA
NADH4L	3445	3723	−	279	21	ATG/TAA
trnT(tgt)	3745	3809	+	65	0	
trnP(tgg)	3810	3874	−	65	26	
NADH6	3901	4404	+	504	3	ATT/TAA
CYTB	4408	5562	+	1155	2	ATG/TAA
trnS2(tga)	5565	5629	+	65	16	
NADH1	5646	6578	−	933	− 47	ATA/TAA
D-loop	6532	6618	+	86	− 33	
trnL1(tag)	6585	6655	−	71	− 29	
rrnL	6627	7972	−	1346	27	
trnV(tac)	8000	8066	−	67	− 1	
rrnS	8066	8827	−	762	57	
Control region	8885	10247	+	1363	64	
trnM(cat)	10312	10379	+	68	3	
trnI(gat)	10383	10449	+	67	− 3	
trnQ(ttg)	10447	10515	−	69	59	
NADH2	10575	11582	+	1008	− 2	ATT/TAA
trnW(tca)	11581	11646	+	66	− 8	
trnC(gca)	11639	11701	−	63	4	
trnY(gta)	11706	11772	−	67	2	
COI	11775	13316	+	1542	− 5	ATT/TAA
trnL2(taa)	13312	13380	+	69	0	
COII	13381	14094	+	714	− 35	ATG/TAA
trnK(ctt)	14060	14130	+	71	− 1	
trnD(gtc)	14130	14197	+	68	0	
ATP8	14198	14359	+	162	− 7	ATT/TAA
ATP6	14353	15030	+	678	8	ATG/TAA
COIII	15039	15827	+	789	2	ATG/TAA
trnG(tcc)	15830	15896	+	67	0	
NADH3	15897	16250	+	354	− 2	ATT/TAG
trnA(tgc)	16249	16313	+	65	0	
trnR(tcg)	16,314	16376	+	63	4	
trnN(gtt)	16381	41	+	68	0	

The length of *L. coffeella* mitogenome (16,407 bp) is larger than *L. malifoliella* (15,646 bp) and in the range of lepidoptera mitogenomes chosen in our study (15,027–17,050 bp) (Table 2). The nucleotide composition of *L. coffeella* is as presented in Table 3 with 41.4%, 40.5%, 7.5% and 10.6% for A, T, G and C, respectively, and is A+T-rich (81.9%) as often described for lepidopteran mitogenomes. The higher bias is in the control region of *L. coffeella* mitogenome (95.6% of A + T), then 84.7% in ribosomal RNAs, 80.9% in transfer RNAs and 79.2% in PCGs.

**Table 2:** The mitochondrial genomes of Lepidoptera selected to reconstruct the phylogenetic trees.

Superfamily	Family	Species	GenBank acess	Size (bp)	Reference
Tineoidea	Tineidae	*Amorophaga japonica*	MH823253	15,027	Kim et al.^51^
Tineoidea	Psychidae	*Mahasena oolona*	NC_036410	16,119	Li et al.^52^
Tineoidea	Psychidae	*Clania variegata*	AP018693	16,601	Arakawa et al.^53^
Geometroidea	Geometridae	*Ectropis obliqua*	NC_036717	16,535	Unpublished
Gelechioidea	Gelechiidae	*Tuta absoluta*	NC_050874	15,290	Yi-Bo et al.^54^
Gracillarioidea	Gracillariidae	*Caloptilia theivora*	NC_046600	15,297	Shin-Chun et al.^55^
Gracillarioidea	Gracillariidae	*Conopomorpha sinensis*	OK310517	17,050	Chang et al.^56^
Gracillarioidea	Gracillariidae	*Gibbovalva kobusi*	MK956103	15,717	Chen et al.^57^
Gracillarioidea	Gracillariidae	*Phyllocnistis citrella*	MN792920	15,416	Liu et al.^58^
Yponomeutoidea	Lyonetiidae	*Lyonetia clerkella*	NC_037944	15,259	Unpublished
Yponomeutoidea	Lyonetiidae	*Leucoptera malifoliella*	JN790955	15,646	Wu et al.^10^
Yponomeutoidea	Praydidae	*Prays oleae*	NC_025948	16,499	Van-Asch et al.^59^
Yponomeutoidea	Attevidae	*Atteva aurea*	NC_067569	16,391	Unpublished
Yponomeutoidea	Scythropiidae	*Scythropia crataegella*	NC_067752	15,350	Unpublished
Yponomeutoidea	Plutellidae	*Acrolepiopsis assectella*	NC_064061	15,369	Unpublished
Yponomeutoidea	Plutellidae	*Plutella xylostella*	JF911819	16,179	Shu-Jun et al.^60^
Yponomeutoidea	Plutellidae	*Plutella australiana*	NC_039687	15,962	Ward and Baxter^61^
Ephydroidea	Drosophilidae	*Drosophila melanogaster*^a^	DMU37541	19,517	Clary et al.^62^

^a^Outgroup: *Drosophila melanogaster.*

**Table 3:** Base composition of genes and control region of the mitochondrial genome of *Leucoptera coffeella*.

Gene/region	Size (bp)	A%	T%	G%	C%	A + T%	AT skew	GC skew
Protein coding genes	11.172	34.0	45.2	10.9	9.9	79.2	− 0.141	0.048
Transfer RNAs	1.467	41.1	39.8	10.8	8.3	80.9	0.016	0.131
Ribosomal RNAs	2.108	42.9	41.8	10.2	5.1	84.7	0.012	0.333
Control region	1.363	46.2	49.4	1.3	3.1	95.6	− 0.033	− 0.409
Complete mitochondrial genome	16.407	41.4	40.5	7.5	10.6	81.9	0.011	− 0.17

The lepidopterans have the second most biased nucleotide composition of the insect orders after Hymenoptera^9^. The AT skew is not significant with 0.011 and the GC skew is moderate with − 0.17 which indicates bias towards the use of As and Cs.

### Protein-coding genes and codon usage

**Figure 2:**
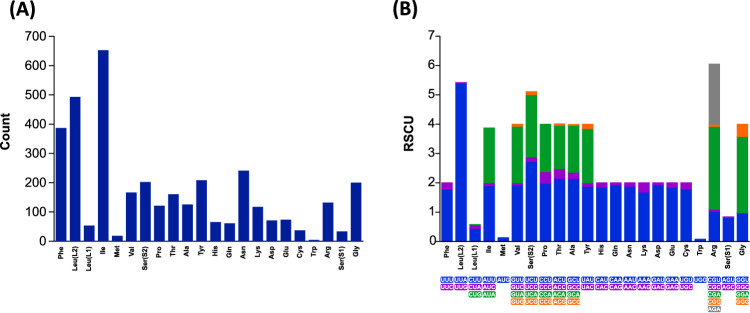
Count (**A**) and frequency values of Relative Synonymous Codon Usage (RSCU) (**B**) in the 13 Protein-Coding Genes of *Leucoptera coffeella* mitochondrial genome.

Thirteen Protein Coding Genes are found in the mitogenome of *L. coffeella* and the average AT content of the PCGs is 79.2%. PCGs displayed nucleotide bias with an AT skew of -0.141 and a GC skew of 0.048 showing that T and G are more abundant than A and C (Table 3). All PCGs use standard ATN start codons and terminate with TAA as codon except for NADH3 using TAG as stop codon (Table 1). Relative synonymous codon usage (RSCU) values for *L. coffeella* are summarized in Table 4 and Fig. 2. NNA and NNT co-dons are more frequent than NNC and NNG indicating a strong A or T bias in the third codon position. The most used amino acids in mitochondrial protein are Ile, Leu and Phe.

**Table 4:** Frequency and RSCU values of relative synonymous codon usage in the 13 protein-coding genes of *Leucoptera coffeella* mitochondrial genome.

Codon	Count	RSCU	Codon	Count	RSCU
UUU(F)	345.00	(1.78)	UAU(Y)	196.00	(1.88)
UUC(F)	42.00	(0.22)	UAC(Y)	12.00	(0.12)
UUA(L)	491.00	(5.40)	UAA(*)	12.00	(1.85)
UUG(L)	2.00	(0.02)	UAG(*)	1.00	(0.15)
CUU(L)	40.00	(0.44)	CAU(H)	60.00	(1.85)
CUC(L)	0.00	(0.00)	CAC(H)	5.00	(0.15)
CUA(L)	12.00	(0.13)	CAA(Q)	59.00	(1.93)
CUG(L)	1.00	(0.01)	CAG(Q)	2.00	(0.07)
AUU(I)	384.00	(1.90)	AAU(N)	227.00	(1.88)
AUC(I)	20.00	(0.10)	AAC(N)	14.00	(0.12)
AUA(I)	249.00	(1.87)	AAA(K)	98.00	(1.68)
AUG(M)	18.00	(0.13)	AAG(K)	19.00	(0.32)
GUU(V)	80.00	(1.93)	GAU(D)	68.00	(1.92)
GUC(V)	3.00	(0.07)	GAC(D)	3.00	(0.08)
GUA(V)	80.00	(1.93)	GAA(E)	67.00	(1.84)
GUG(V)	3.00	(0.07)	GAG(E)	6.00	(0.16)
UCU(S)	108.00	(2.73)	UGU(C)	33.00	(1.78)
UCC(S)	6.00	(0.15)	UGC(C)	4.00	(0.22)
UCA(S)	84.00	(2.13)	UGA(*)	91.00	(1.92)
UCG(S)	4.00	(0.10)	UGG(W)	4.00	(0.08)
CCU(P)	60.00	(1.98)	CGU(R )	13.00	(1.02)
CCC(P)	12.00	(0.40)	CGC(R)	1.00	(0.08)
CCA(P)	49.00	(1.62)	CGA(R)	36.00	(2.82)
CCG(P)	0.00	(0.00)	CGG(R)	1.00	(0.08)
ACU(T)	86.00	(2.15)	AGU(S)	32.00	(0.81)
ACC(T)	13.00	(0.33)	AGC(S)	1.00	(0.03)
ACA(T)	59.00	(1.48)	AGA(R)	81.00	(2.05)
ACG(T)	2.00	(0.05)	AGG(R)	0.00	(0.00)
GCU(A)	67.00	(2.14)	GGU(G)	48.00	(0.96)
GCC(A)	7.00	(0.22)	GGC(G)	1.00	(0.02)
GCA(A)	50.00	(1.60)	GGA(G)	130.00	(2.60)
GCG(A)	1.00	(0.03)	GGG(G)	21.00	(0.42)

A total of 3724 codons were analyzed, excluding the initiation and termination codons.

### Overlapping and intergenic spacer regions

Six tRNAs (trnT (TGT), trnL2 (TAA), trnD (GTC), trnG (TCC), trnA (TGC) and trnN (GTT)) do not have intergenic nucleotides. Twenty intergenic spacer regions (Table 1) of a total of 381 nucleotides were identified, ranging from 2 to 64 nt. The latter is located between the control region and trnM. Control region, trnQ (TTG), rrnS, and COII are the features with the higher intergenic spacer regions size with 64 nt, 59 nt, 57 nt, and 35 nt, respectively (Table 1). Region overlaps from 1 to 47 nt were observed for eleven gene pairs (Table 1). One of these regions overlaps is a 7 bp motif (ATGATAA) found in *L. coffeella* mitogenome between ATP8 and ATP6. This motif was found at the same position in another Yponomeutidae mitogenome^23^ but also in Danaidae^24,25^ and Coleoptera genomes^25,26^. It is also found in our mitogenome at the beginning of NADH4 and NADH4L genes as well as in the coding regions of 4 other genes (rrnL, rrnS, NADH2 and trnN).

### Transfer RNA genes (tRNA) and Ribosomal RNA genes

*L. coffeella* mitogenome contains 22 tRNAs, one for each of the 20 amino acids with an additional isotype for each of the two sixfold degenerate amino acids Leucine and Serine. Seven of these tRNAs (trnF, trnH, trnP, trnV, trnQ, trnC, trnY) are coded by the minority strand (Table 1) while the other fifteen tRNA genes are encoded by the majority strand.

The total length of the 22 tRNA is 1467 bp with lengths ranging from 61 to 71 bp and their A-T content is 80.9%. The AT skew is slightly positive, 0.016 and the CG skew is positive, 0.131 (Table 3).

**Figure 3:**
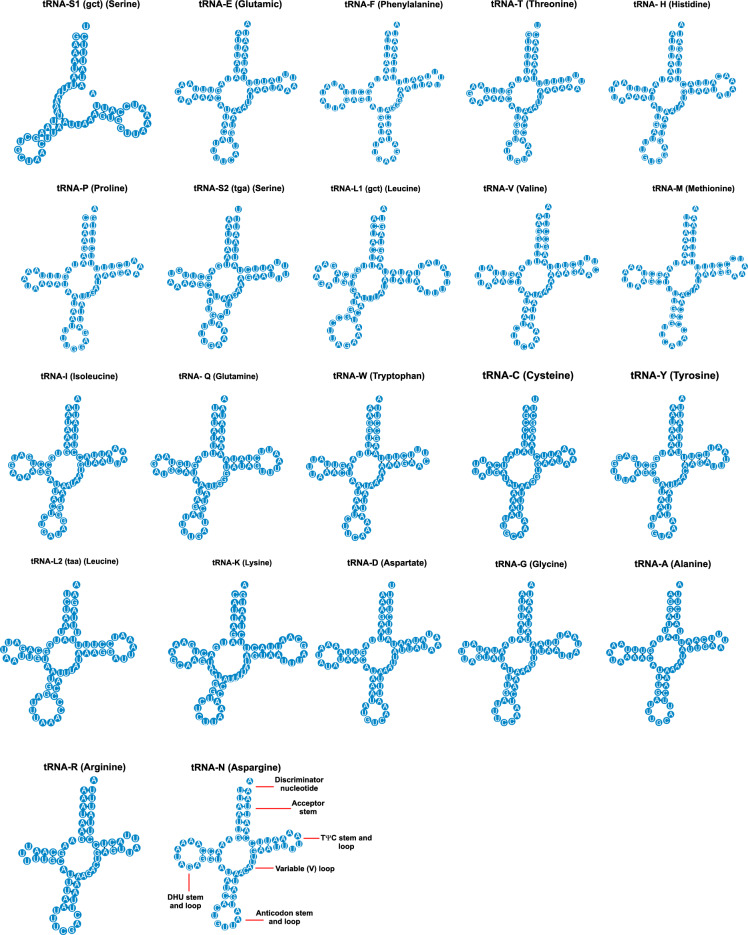
Secondary cloverleaf structure for the 22 tRNAs of *Leucoptera coffeella* mitochondrial genome.

The clover-leaf structure is found for all the 22 tRNAs from *L. coffeella* with the exception of trnS1 and trnS2 (Fig. 3). The trnS1 is missing the dihydrouridine (DHU) arm replaced by an unstable loop while the trnS2 has an additional loop in the anticodon-stem and loop. These two tRNAs are found on the minority strand. Lack of the DHU stem-loop in trnS1 is nearly ubiquitous in insect mitochondrial genomes^9^.

The 16S RNA (rrnL) is located between tnrL1 and trnV and its length is 1345 bp whereas the 12S RNA (rrnS), located between trnV and the control region, is 761 bp. The A + T content of the two rRNAs genes is 84.7% (Table 3).

### *The A* + *T-rich region (control region)*

The mitochondrial genome of *L. coffeella* contains a 1363 bp A + T-rich region or control region located between rrnS and trnM genes (Table 1). It is one of the largest A + T-rich region found in the Lepidoptera order, and with the highest A + T content, 95.6% (Table 3). This control region includes initiation sites for transcription and replication.

**Figure 4:**
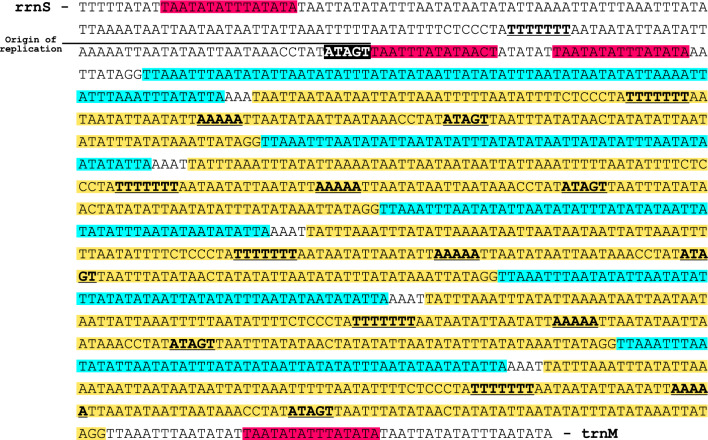
Control region of *Leucoptera coffeella* mitochondrial genome. In red: 15 nt repeat, in blue: 57 nt repeat, in yellow: 159 nt repeat, in black: origin of replication. Poly-T and poly-A stretches are in bold and underlined. rrnS and trnM are the genes surrounding the control region.

The structure of the A + T-rich region of *L. coffeella* is composed of five tandem repeats elements and a motif containing the origin of replication 'ATAGT' (Fig. 4). We found five repeats composed of a 57 bp (in blue) and a 159 bp (in yellow) sequences, and four (TA)n microsatellite regions (in red) (Fig. 4). Five Poly(T)7 and Poly(A)5 were also found but these repetitions are shorter compare to other lepidopteran’s control regions^22,23^. The A + T-rich region of *L. coffeella* mitochondrial genome presents differences compared to other Lyonetiidae, such as *L. malifoliella* mitogenome which has a shorter control region of 733 bp and the ATAGA motif (Fig. S1)^10^. We did not find the poly-T stretch downstream of the rrnS gene that is widely conserved in lepidopteran mitogenome*. L. coffeella* control region is also missing the poly-A stretch immediately upstream of the trnM gene, a feature commonly observed in other lepidopteran mitogenomes including *L. malifoliella*. Both *L. malifoliella* and *L. coffeella* have a stem-loop structure in the control region (Fig. S2). Such a feature seems to be intrinsic to the control region of *Leucoptera* species^10^.

The presence of a stable stem-loop structure in the A + T-rich region of *Leucoptera* appears to be as important as the presence of a stretch poly-T microsatellite in other insects, which, unless associated with a recognition of the light stretch origin of replication, has not yet been fully explored^27^.

The lack of molecular data for other *Leucoptera* species limits this interpretation and reinforces the need to expand the sampling sizes and deepen our understanding of the replication and transcription origin of the mt genome of *Leucoptera* species.

### Gene rearrangements

We compare *L. coffeella* gene order to insect and Lepidoptera ancestral gene rearrangements^9^ in order to identify possible reorganization such as duplication, deletion, or inversion-translocation. We analyzed gene rearrangements using qMGR program^28^.

**Figure 5:**
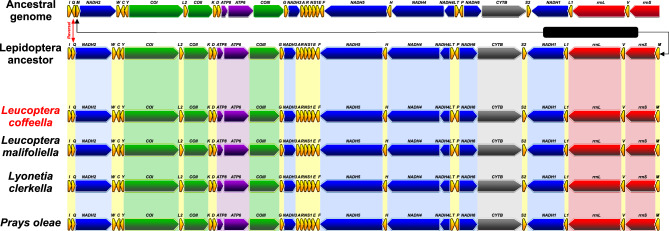
Comparison of gene rearrangement for *Leucoptera coffeella* mitochondrial genome (in red) with insect Ancestral genome, Lepidoptera ancestor, *L. malifoliella*, *L. clerkella* and *P. oleae* mitogenomes.

*L. coffeella* has exactly the same genes order than the two other Lyonetiidae mitogenomes available (Fig. 5). It is also identical to the gene order model proposed for ancestral Lepidoptera mitogenome which exhibits the trnM, trnI, trnQ (MIQ) common rearrangement with trnM on the minority strand. MIQ is found in most ditrysians between the A + T rich region and NADH2^9^. *L. coffeella* also shares the A-R-N-S1-E-F gene rearrangement of insect ancestor between NADH3 and NADH5. *L. coffeella* mitogenome gene arrangement is identical to the ancestral Lepidoptera mitogenome organization proposed by Cameron^9^ and Moreno-Carmona et al.^29^.

### Phylogenetic relationships

**Figure 6:**
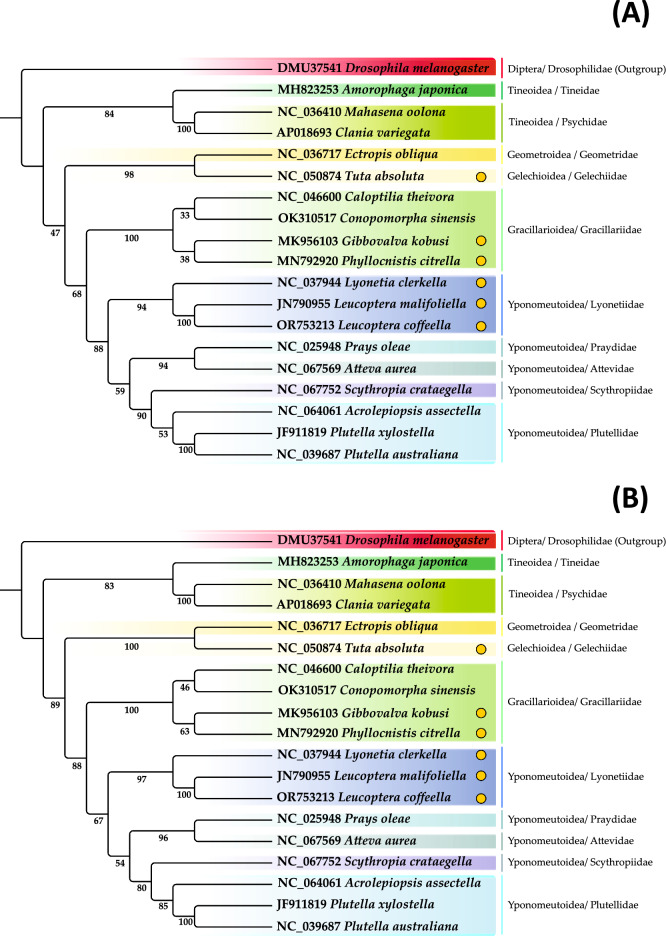
Phylogenetic trees of *Leucoptera coffeella* using the 13 concatenated PCGs using (**A**) nucleotide sequences and (**B**) amino acid sequences data. Bootstrap values are indicated on each branch. Maximum-Likelihood method was used. The yellow circle indicates the lepidopteran’s leaf miners.

The phylogenetic analysis was performed with the ML method using 13 concatenated PCG from 18 lepidopteran species and one Diptera (Table 2; Fig. S3). The topologies of the phylogenetic trees were identical using either nucleotides (Fig. 6B) or amino acids data (Fig. 6B). A backbone in the phylogenetic tree was found: ((((((Lyonetiidae), ((Plutellidae, Scythropiidae), (Praydidae, Attevidae))), Gracillariidae), (Gelechiidae, Geometridae)), (Tineidae, Psychidae)), (Drosophilidae)).

All Yponomeutoidea’s families form a monophyletic clade and are found in a clade with the Gracillariidae from Gracillarioidea. The Gracillarioidea, with Gracillariidae, Psychidae and Tineidae, forms a polyphyletic group as it was previously described in a phylogenetic study based on 794 lepidopteran mitogenomes^30^. Within Yponomeutoidea, Praydidae and Attevidae form a sister group (PrAt) with a high BS of 96. Plutellidae and Scythropiidae from a sister group (PlSc) with a BS of 80. We obtained better BS values for these four relationships compared to another phylogenetical study recently published^31^. The alignment of our sequences after concatenation using mafft might explain the improvement of nodal values (se Fig. S3). However, we did not improve the nodal support between PrAt and PlSc (54) and in this case more data are needed. Gracillariidae and Yponomeutoidea are present in the same clade with bootstrap value of 88. Leaf-mining feeding behaviors was characterized in Gracillariidae as the most phylogenetically conserved trait^32^.

A most recent study including 130 gracillariid species linked mining as an ancestral larval behavior of Gracilllariidae that has evolved several times^33^. All or part of the larval period as a leaf-miner might confer ecological advantage such as protection from natural enemies (predators, parasitoids, pathogens), from variation in their environment (UV radiation, hygrothermy)^34^. Another hypothesis is that leaf miners fed selectively on the most nutritious layers of foliage tissue avoiding plant defenses*. L. coffeella* is found in the same clade as *L. malifoliella* and *L. clerkella*, two other leaf miners of the Lyonetiidae family with bootstrap of 100 and 97 for nucleotides and amino acids phylogenies, respectively. The Lyonetiidae family includes leaf miners considered as agronomic pests, however, insufficient molecular data is limiting the phylogenetic inferences about this family.

Only the three Lyonetiidae species included in our phylogeny have been sequenced. We only observed the presence of leaf miners which diverged in their host-plant preferences. *L. coffeella* feeds exclusively on coffee plants^35^.

*L. malifoliella* is polyphagous^36^ and *L. clerkella* feeds on *Malus* sp. and *Prunus* sp. species^37^. In Yponomeutoidea, the large proportion of representatives are oligophagous^38^. Further molecular studies of lepidopteran leaf miners are needed to better understand how this feeding habit innovation occurs in Lyonetiidae and Gracillariidae. The host plant range in these two families should also be further study to confirm the preference for shrubs and trees^34,38, 39^.

## Conclusions

Mitochondrial genomes sequences are increasingly used as informative molecular markers for systematics, phylogenetics, population genetics and evolutionary studies because of its conserved gene content, its small size, its fast rate of evolution, its minimal or absent sequence recombination, its maternal inheritance and its abundant markers types. Here we report the first complete mitochondrial genome of *L*. *coffeella*. It consists of a circular double stranded DNA of 16,407 bp containing the conserved trnM-trnI-trnQ gene rearrangement found in Lepidoptera ancestors^9^.

We found 22 tRNAs showing conserved clover leaf structure, except for the trnS1 and trnS2 coding for serine tRNAs. We also observed a codon usage bias, with high variability detected in the third position of codons. Regarding the most closely related mitogenome, the main difference between *L. coffeella* and *L*. *malifoliella* is in the control region size, with 1363 bp and 733bp, respectively.

Our phylogenetic study based on Maximum-likelihood estimation confirms the presence of *L*. *coffeella* and *L. malifoliella* in the Lyonetiidae clade and in the Yponomeutoidea superfamily with insects from Plutellidae and Praydidae. Our phylogeny points out that the leaf-mining habit was acquired several times through the evolution of Lepidoptera, as we can for instance found leaf-miners in both the Lyonetiidae family as well as in Gracillariidae clades. The acquisition of this innovation was followed by host-plant specialization with *L*. *coffeella* on coffee tree, *L*. *clerkella* on *Malus* and *Prunus,* and *L*. *malifoliella* being polyphagous.

Acquisition of more genomic data in this part of the tree is needed to confirm this hypothesis. We have acquired molecular data that can now be used to learn more about the history of *L*. *coffeella* introduction and its invasive route in the Neotropical Americas. How this insect has adapted to the conditions of coffee crops in these regions might help the development of Integrated Pest Management programs and the use of for instance parasitoids.

## Materials and methods

### Ethics statement

Our study did not involve any endangered or protected species. No specific permits were required for the insect or plant specimen collection in this study. The collection and use of plant and insect materials in the study comply with relevant institutional, national, and international guidelines and legislation.

### Leucoptera coffeella genomic DNA

Genomic DNA (gDNA) was obtained from a pool of individuals at the pupae stage feeding on *Coffea arabica* (L.) (Rubiaceae) leaves at latitude − 15.72812S; longitude − 47.90277W, Brasilia-DF (Brazil). High-quality gDNA samples were extracted as described in^40^ and sequenced using PacBio HiFi technology (DNA Link Sequencing Lab DNALINK, Seoul, Republic of Korea). The sequencing was performed using PacBio HiFi technology. The genome assembly is described by Martins et al.^21^, and is available at GenBank BioProject ID PRJNA832598^21^.

### Genome assembly and annotation

We used two mitochondrial genes fragments from *L. coffeella* available in GenBank, COI (MF987402) and Cytb (MF987470) and the orthologous genes from *L. malifoliella*, COI (GU929715) and Cytb (NC_018547) to search the mitochondrial sequences present in the genome assembly (PRJNA832598). From the blastn search, we retrieved one contig containing two matches for each gene query containing COI and Cytb. To assemble the whole mitochondrial genome of *L. coffeella*, we used the raw reads used for the genome assembly and submitted the raw reads to Aladin v.3.0 software (https://github.com/GDKO/aladin) using the *L. malifoliella* mitochondrial genome as seed^10^.

We annotated the mitogenome sequence obtained from Aladin with MITOS2 web-server (http://mitos.bioinf.uni-leipzig.de/index.py)^41^ with reference ‘RefSeq 81 Metazoa’ invertebrate ‘5’ genetic code. The mitogenome data from *L*. *coffeella* was deposited in Genbank at NCBI (submission ID: BankIt2758416 OR753213, Supplementary data).

### Bioinformatics analysis

The nucleotide base composition was determined using ‘wordcount’ program of the EMBOSS toolkit v. 6.6.0.0^42^, and the AT/CG skewness was calculated using the formula AT skew = [A − T]/[A + T] while GC skew = [G − C]/[G + C]^43^. The tRNA genes, their secondary structures, the gene overlapping, and intergenic spacers were predicted using MITOS2.

The tandem repeats in the control region were located using MEME suite (https://meme-suite.org/meme/)^44^. MEME version 5.5.3 was used to search for repeat motifs between 6 to 300 nt within the control region of *L*. *coffeella* or *L*. *malifoliella*. The comparison of *L*. *coffeella* mitochondrial genome gene order with the Lepidoptera ancestor’s rearrangement (trnI-trnQ-trnM)^9^ was performed using the program qMGR program^28^. The Relative Synonymous Codon Usage (RSCU) of PCGs was determined using MEGA11^45^. The representation of the mitogenome circular map was done with the web tool OGDRAW v.1.3.1^46^ by MPI-MP CHLOROBOX.

### Phylogenetic analysis

We aligned 18 mitochondrial Lepidopteran genomes of Yponomeutoidea, Tineoidea, Gracillarioidea; Gelechioidea, Geometroidea and Ephydroidea species (Table 2) to reconstruct the phylogenetic trees, with *Drosophila melanogaster* as the outgroup. The nucleotide sequences of the selected species were downloaded from NCBI database (https://www.ncbi.nlm.nih.gov, June 2023). We emphasized in taxon sampling some Lepidopteran’s leaf miner species with assembled and annotated complete mitochondrial genomes publicly available in GenBank.

The 13 PCG genes (COI, COII, COIII, Cytb, ATP6, ATP8, NADH1-NADH6 and NADH4L) of each species were translated to amino acid sequences using TransDecoder v.5.5.0 [https://github.com/TransDecoder/TransDecoder] and concatenated with the perl script FASconCAT v.1.11^47^. The concatenated PCG sequences were globally and locally aligned using the program MAFFT v7.475^48^ with the Needleman-Wunsch algorithm for nucleotide sequences (mafft G-INS-i) and Smith-Waterman for amino acid sequences (mafft L-INS-i) and 1000 maximum refinement interactions. The amino-acid and nucleotidic alignments are available at 10.57745/NA3OZ2. The evolutionary models for phylogenetic trees were determined using ModelTest-NG^49^. The GTR substitution model with gamma-distributed rate variation and proportion of invariable sites (GTR + I + G4) was selected for nucleotide sequences, (BIC score: 20,6910.5687 and weight: 1.0000), and the model MTART + I + G4 + F was selected for amino acid sequences (BIC score: 11,1197.8918 and weight: 1.0000). The Maximum-likelihood analysis was performed with RAxML-NG v. 0.9.0^50^ with 1000 of bootstrap replicates.

Figure S3 is the bioinformatic pipeline of the *Leucoptera coffeella* mitochondrial genome obtention. The amino-acid and nucleotide alignments used for the phylogeny of Fig. 6 can be found at 10.57745/NA3OZ2.

### Supplementary Information

Supplementary Information.

## Data Availability

The datasets generated and analyzed during the current study are available in the NCBI repository (BankIt2758416 OR753213).
