# Supplementary material for: The complete mitochondrial genome of Leucoptera coffeella (Lepidoptera: Lyonetiidae) and phylogenetic relationships within the Yponomeutoidea superfamily
Source: Sci Rep. 2024 Mar 26;14:7119. doi: 10.1038/s41598-024-57869-3 (PMC10965899; doi:10.1038/s41598-024-57869-3)
Supplement: Supplementary file 1 — Supplementary Information. [file 41598_2024_57869_MOESM1_ESM.docx]

**The complete mitochondrial genome of *Leucoptera coffeella* (Lepidoptera: Lyonetiidae) and phylogenetic relationships within the Yponomeutoidea Superfamily**

**Supplementary data**



**Figure 1S.** Statistics of the control region of genome of *Leucoptera coffeella* (a) and *L. malifoliella*.


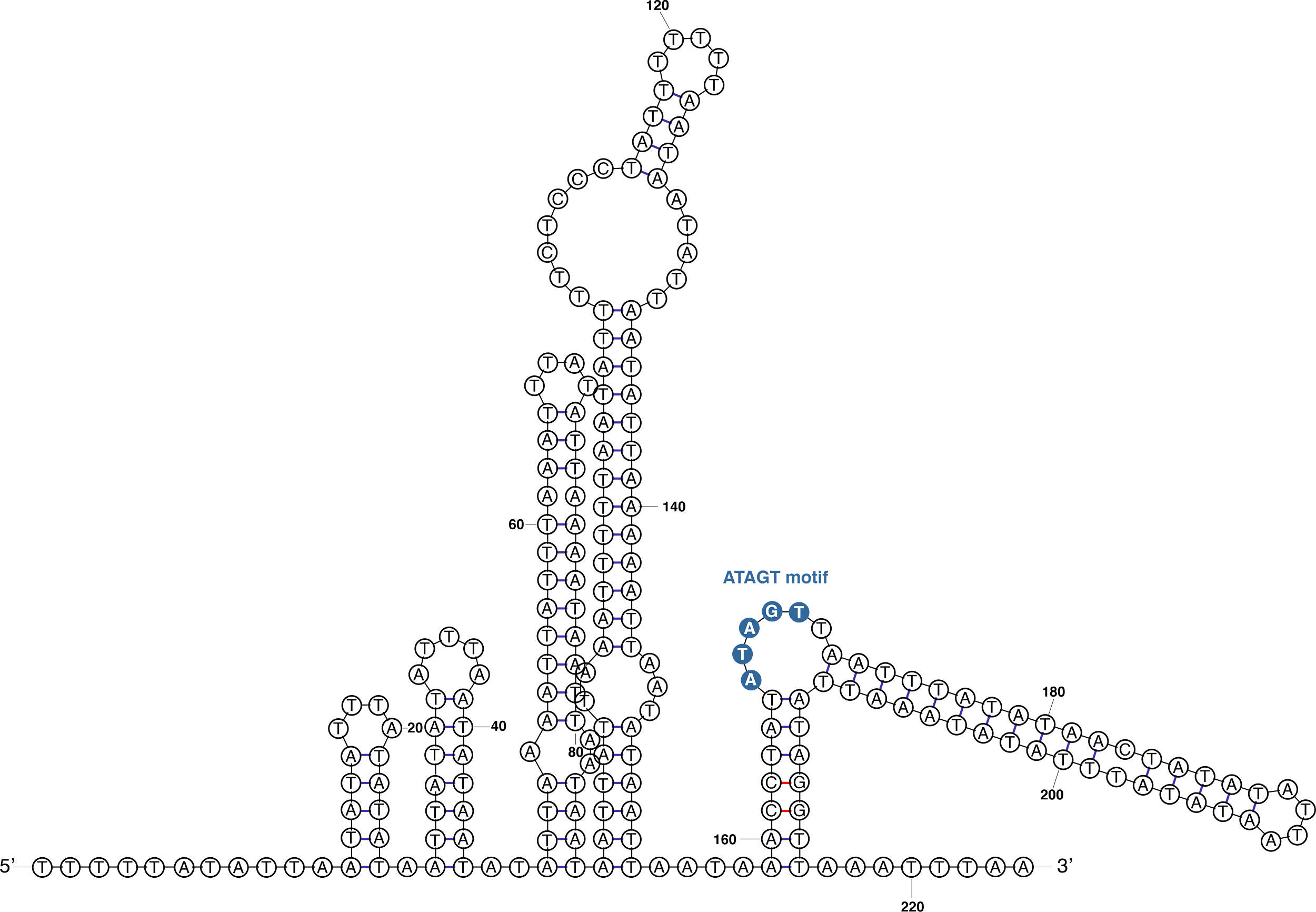


**Figure 2S.** Structure of the control region of genome of *Leucoptera coffeella*.


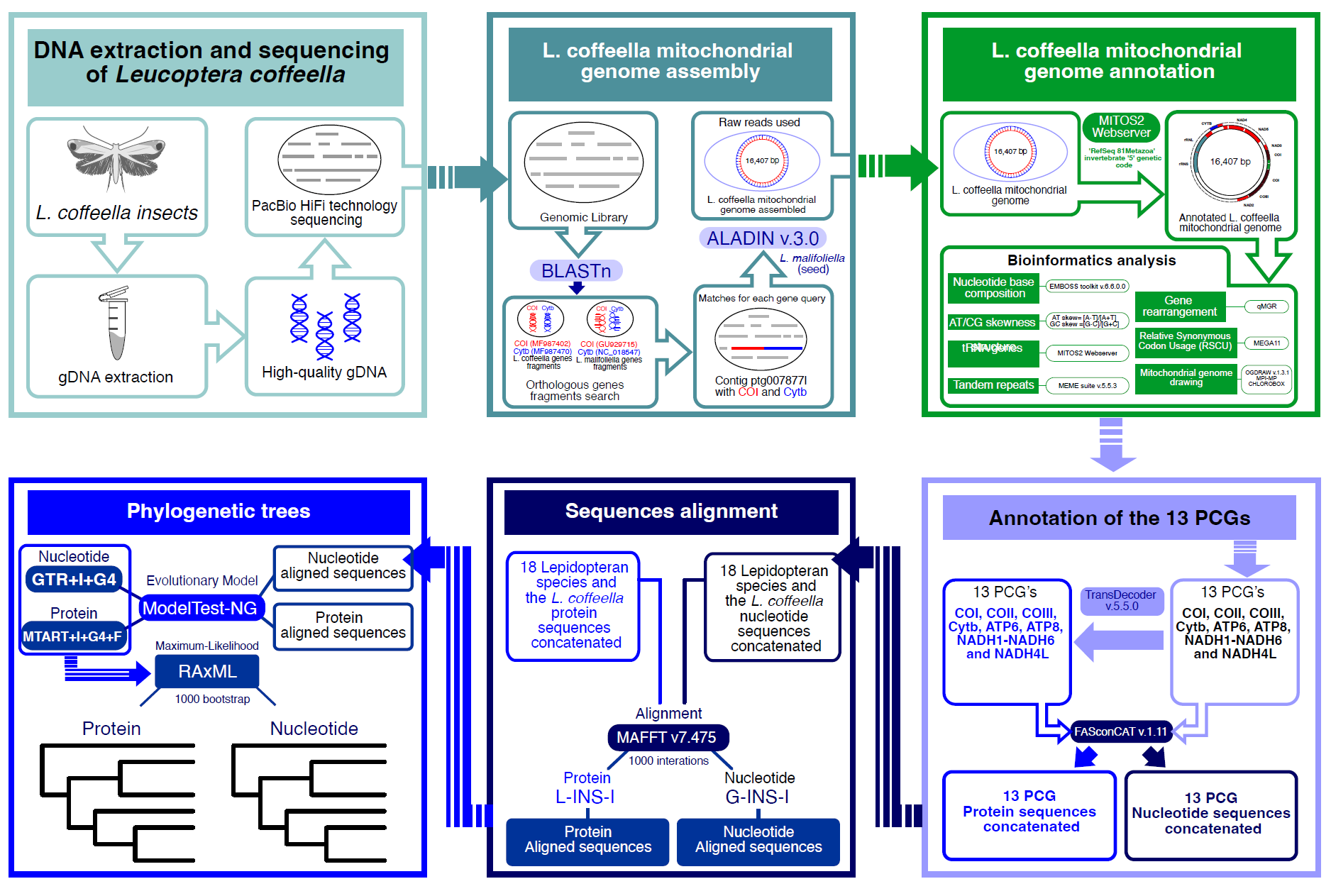


**Figure 3S.** Bioinformatic Pipeline used to obtain *Leucoptera coffeella* mitochondrial genome and the phylogenies presentedn in the manuscript.

**NCBI_mitogenome_Leucoptera_coffeella_Submission BankIt2758416 OR753213**

LOCUS Seq1 16407 bp DNA circular INV 27-OCT-2023

DEFINITION Leucoptera coffeella mitochondrion, complete genome.

ACCESSION Seq1

VERSION

KEYWORDS .

SOURCE mitochondrion Leucoptera coffeella

ORGANISM Leucoptera coffeella

Eukaryota; Metazoa; Ecdysozoa; Arthropoda; Hexapoda; Insecta;

Pterygota; Neoptera; Endopterygota; Lepidoptera; Glossata;

Ditrysia; Yponomeutoidea; Lyonetiidae; Leucoptera.

REFERENCE 1 (bases 1 to 16407)

AUTHORS Santos,M.P., Mota,A.P.Z., Togawa,R.C., Martins,N.F.,

Nascimento,E.F.M.B., Lucena-Leandro,V.S., Castellani,M.A.,

Albuquerque,E.V.S. and Hilliou,F.

TITLE The complete mitochondrial genome of Leucoptera coffeella

(Lepidoptera: Lyonetiidae) and phylogenetic relationships within

the Yponomeutoidea Superfamily

JOURNAL unpublished

REFERENCE 2 (bases 1 to 16407)

AUTHORS Santos,M.P., Mota,A.P.Z., Togawa,R.C., Martins,N.F.,

Nascimento,E.F.M.B., Lucena-Leandro,V.S., Castellani,M.A.,

Albuquerque,E.V.S. and Hilliou,F.

TITLE Direct Submission

JOURNAL Submitted (27-OCT-2023) Crop Science and Animal Science, State

University of Southwestern Bahia, Estrada do Bem Querer, Vitoria da

Conquista, Bahia 3293-3391, Brazil

COMMENT Bankit Comment: ALT EMAIL:2022f0072@uesb.edu.br

Bankit Comment: TOTAL # OF SEQS:1

##Assembly-Data-START##

Assembly Method :: Aladin v. v.3.0

Assembly Name :: Ana P. Z. Mota

Coverage :: 100

Sequencing Technology :: PacBio

##Assembly-Data-END##

FEATURES Location/Qualifiers

source 1..16407

/organism="Leucoptera coffeella"

/organelle="mitochondrion"

/mol_type="genomic DNA"

/host="Coffea arabica"

/db_xref="taxon:1178041"

/country="Brazil"

/lat_lon="-15 degrees 43' 41,232';-47 degrees 54' 09,972'"

/collected_by="E.V.S. Albuquerque, E. F.M.B. Nascimento,

V.S.Lucena-Leandro"

tRNA join(1..41,15381..16407)

/product="tRNA-Asn"

/note="trnN(gtt)"

/anticodon=(pos:5..7,aa:Asn,seq:gtt)

gene 42..5629

/gene="tRNA-Ser"

tRNA 42..102

/gene="tRNA-Ser"

/product="tRNA-Ser"

/note="trnS1(gct)"

/anticodon=(pos:63..65,aa:Ser,seq:gct)

/gene="tRNA-Ser"

gene 107..172

/gene="tRNA-Glu"

tRNA 107..172

/gene="tRNA-Glu"

/product="tRNA-Glx"

/note="trnE(ttc)"

/anticodon=(pos:137..139,aa:Glx,seq:ttc)

/gene="tRNA-Glu"

gene complement(178..245)

/gene="tRNA-Phe"

tRNA complement(178..245)

/gene="tRNA-Phe"

/product="tRNA-Phe"

/note="trnF(gaa)"

/anticodon=(pos:complement(210..212),aa:Phe,seq:gaa)

/gene="tRNA-Phe"

gene complement(273..1985)

/gene="NADH5"

/note="Start codon ATT/ final codon TAA"

CDS complement(273..1985)

/gene="NADH5"

/codon_start=1

/transl_table=5

/product="NADH dehydrogenase subunit 5"

/translation="MCFNSFFFLFFFSLMNFFFFIYFMIKNLVLFIEWEVFSFNSNVV

YMSILIDWMSLLFMSFVLLISSVVIKYSNSYMESDKNLVRFILLVLLFVFSMMLLIIS

PNLISILLGWDGLGLISYCLVIYYQSYKSYNSGMLTALSNRIGDVFILICISWMLNYG

SWNYIFYLNFMKNDFSMQMISFMIVIAAMTKSAQIPFSSWLPAAMAAPTPVSALVHSS

TLVTAGVYLLIRFSDLLMGMYVLKFLLLLSTLTMFMAGIVANYEFDLKKIIAFSTLSQ

LGLMMSVLCMGFPKLAFFHLLTHAMFKALLFMCAGLIIHMMFDMQDIRYMGGMCKYIP

LTSLCMVISNLSLCGLPFMSGFYSKDLMLDSISMMNINIFIYVIFYLSTGLTVFYTFR

MLFYLMISDFNLVSIYNLFEEDYNMIYSMFVLLFMSLISGSMLSWLLFYDVNIIYMFL

HMKIMVIYVSLVGMLLGYLVSLFNYNFLNKFMLTYKISFFFSTMWFMVGLSTYCINYN

FLKFGQILYKNDRGWVEYLSGQGLFNTLKFYSIFYYFFQLNNLKIYLFSFVLWMMIYI

MIII*"

gene complement(2001..2068)

/gene="tRNA-His"

tRNA complement(2001..2068)

/gene="tRNA-His"

/product="tRNA-His"

/note="trnH(gtg)"

/anticodon=(pos:complement(2036..2038),aa:His,seq:gtg)

/gene="tRNA-His"

gene complement(2097..3437)

/gene="NADH4"

/note="Start codon ATG/ final codon TAA"

CDS complement(2097..3437)

/gene="NADH4"

/codon_start=1

/transl_table=5

/product="NADH dehydrogenase subunit 4"

/translation="MMKFFLFLLFMMPLCFFFNMFWMVQMMMFLLFFLFLLNYISLGG

MYSISYGFGCDMMSYGLILLSIWVCCLMITSSESLYKLNFFLNGFLINLIILMVMLML

TFSTMNLFMFYFFFEGSLIPTLMLIVGWGYQPERMQAGLYLLFYTLFASLPMLMGIFF

LYKNLNCLSFYMLKFYNFTFFYLYIVMILAFLVKMPMYFVHLWLPKAHVEAPISGSMI

LAAIMLKLGGYGLMRVMIFLQEISVKLSFIWIIISLIGGVYISLVCLVQIDIKALIAY

SSVAHMSLVIGGIMTMNYWGFMGSYIMMIGHGLCSSGMFCLSNMNYERLSSRSLLVNK

GMMNFMPSMSMWWFLLLSSNMSCPPSLNLAGEISLINSLLSWSWLSMFLLMLVSFFSA

AYSLYLYSYTQHGMIFKGIYSFYSGTSREYLLLLLHWLPLNLLILKVDYFLIWI*"

gene complement(3445..3723)

/gene="NADH4L"

/note="Start codon ATG/ final codon TAA"

CDS complement(3445..3723)

/gene="NADH4L"

/codon_start=1

/transl_table=5

/product="NADH dehydrogenase subunit 4L"

/translation="MMKMIFFMFFIGNMIFVSKYKHLMIVLLSLEYLVLSIFIFLLVF

LMYYNCDMYMLMVFLVFSVCEGVLGLSVLVSMIRSHGNDYFQSFNLLN*"

gene 3745..3809

/gene="tRNA-Thr"

tRNA 3745..3809

/gene="tRNA-Thr"

/product="tRNA-Thr"

/note="trnT(tgt)"

/anticodon=(pos:3775..3777,aa:Thr,seq:tgt)

/gene="tRNA-Thr"

gene complement(3810..3874)

/gene="tRNA-Pro"

tRNA complement(3810..3874)

/gene="tRNA-Pro"

/product="tRNA-Pro"

/note="trnP(tgg)"

/anticodon=(pos:complement(3842..3844),aa:Pro,seq:tgg)

gene 3901..4404

/gene="NADH6"

/note="Start codon ATT/ final codon TAA"

CDS 3901..4404

/gene="NADH6"

/codon_start=1

/transl_table=5

/product="NADH dehydrogenase subunit 6"

/translation="MINLSFLMYFLNHPLTMGFMIIVQTLLTCLMSGLLLNTYWFSYI

LFLTFLGGMLVLFIYVSSIASNELFYNSKYLLMMLIMNFFLLFLMYIIFYNKEMEIKN

INNEMNKLNYEFFMFNYEYKINISKMYNNQSPFMMYMLIIYLFITLIAVVKINNIFYG

PLRSSNF*"

gene 4408..5562

/gene="CYTB"

/note="Start codon ATG/ final codon TAA"

CDS 4408..5562

/gene="CYTB"

/codon_start=1

/transl_table=5

/product="Cytochrome b"

/translation="MKNNFKSILKTHPILKIINGSLINLPSPSNISIWWNFGSLLGLC

LMIQIITGLFLTMYYTANIDMAFFSVNYICRNVNYGWLIRTMHANGASLFFICIFLHI

GRGMYYESFILKVTWLVGTLILFILMATAFLGYVLPWGQMSFWGATVITNLLSAIPYL

GNTLVVWIWGGFAIDNATLTRFYTFHFILPFVILMMSMIHLMFLHQTGSNNPLGIKSN

MDKIPFHPFFTWKDILGFIVVIFLFLMLVLINPNYLGDPDNFIPANPLVTPAHIKPEW

YFLFAYAILRSIPNKLGGVIALIFSILILMTLPFTHLKKTQGIQFYPLNQIYFWSIVV

TLILLTWIGARPVEDPYITTGQILAIYYFSYFIFNPLLGKLWDDYLFKYN*"

tRNA 5565..5629

/gene="tRNA-Ser"

/product="tRNA-Ser"

/note="trnS2(tga)"

/anticodon=(pos:5594..5596,aa:Ser,seq:tga)

/gene="tRNA-Ser"

gene complement(5646..6578)

/gene="NADH1"

/note="Start codon ATA/ final codon TAA"

CDS complement(5646..6578)

/gene="NADH1"

/codon_start=1

/transl_table=5

/product="NADH dehydrogenase subunit 1"

/translation="MIKDNLMIFFSLLVLFIGVLIGVAFLTLLERKLLGYIQFRKGPN

KVGLMGILQPFSDGIKLFTKEQTYPMYSNFIVYYFSPVFSFLFSLIIWLLIPYYFNLV

SFNLGMLFFFCVTSMGVYTVMLAGWSSNSNYALLGGLRSVAQTISYEVSLVLIIMSSI

ILLMDFGLFNFMIYQKFMWFMIYMIPLSLMMISSMLAETNRTPFDFAEGESELVSGFN

IEYSSGGFALIFLAEYSSILFMSMLFVLLYMGGYSLYLFYFKLTFISFLFVWVRGTLP

RYRYDKLMYLAWKIYLPVSLNFLLFFVGLKIMFI*"

D-loop 6532..6618

/note="D-loop"

gene complement(6585..6655)

/gene="tRNA-Leu"

tRNA complement(6585..6655)

/gene="tRNA-Leu"

/product="tRNA-Leu"

/note="trnL1(tag)"

/anticodon=(pos:complement(6624..6626),aa:Leu,seq:tag)

gene complement(6627..7972)

/gene="16S ribosomal RNA"

rRNA complement(6627..7972)

/gene="16S ribosomal RNA"

/product="16S ribosomal RNA"

/gene="16S ribosomal RNA"

gene complement(8000..8066)

/gene="tRNA-Val"

tRNA complement(8000..8066)

/gene="tRNA-Val"

/product="tRNA-Val"

/note="trnV(tac)"

/anticodon=(pos:complement(8035..8037),aa:Val,seq:tac)

/gene="tRNA-Val"

gene complement(8066..8827)

/gene="12S ribosomal RNA"

rRNA complement(8066..8827)

/gene="12S ribosomal RNA"

/product="12S ribosomal RNA"

/gene="12S ribosomal RNA"

C_region 8885..10247

/note="A+T rich region"

gene 10312..10379

/gene="tRNA-Met"

tRNA 10312..10379

/gene="tRNA-Met"

/product="tRNA-Met"

/note="trnM(cat)"

/anticodon=(pos:10343..10345,aa:Met,seq:cat)

/gene="tRNA-Met"

gene 10383..10449

/gene="tRNA-Ile"

tRNA 10383..10449

/gene="tRNA-Ile"

/product="tRNA-Ile"

/note="trnI(gat)"

/anticodon=(pos:10413..10415,aa:Ile,seq:gat)

/gene="tRNA-Ile"

gene complement(10447..10515)

/gene="tRNA-Gln"

tRNA complement(10447..10515)

/gene="tRNA-Gln"

/product="tRNA-Gln"

/note="trnQ-(ttg)"

/anticodon=(pos:complement(10483..10485),aa:Gln,seq:ttg)

/gene="tRNA-Gln"

gene 10575..11582

/gene="NADH2"

/note="Start codon ATT/ final codon TAA"

CDS 10575..11582

/gene="NADH2"

/codon_start=1

/transl_table=5

/product="NADH dehydrogenase subunit 2"

/translation="MFINSNKMFFLFILMISTMISISSNSWFGCWIGLEINLLSFIPL

ISNSKNLLSSEASLKYFLTQSIASINLLFLIIMMSFFKQLENYNIMMLLMNLPLLMKM

GSAPFHFWFPNLIEGLSWMNAYIIMTWQKITPLILISYNYNFYLISFIAILNAFVGAI

GGFNQMSLRKILTFSSINNLSWMLISIMISENLWMLYFFIYCILNFMLCYFFNLMNIF

YINQIYFYNMKNFYKILLAMNLFSLGGLPPFMGFLSKWLVINFLINNNMLMLTFLMIM

SSLIMLYYYIRIMYSTFLINYMKLKWFNFKIKNNFNKYYFICMYMFSFLGIPISTLTF

F*"

gene 11581..11646

/gene="tRNA-Trp"

tRNA 11581..11646

/gene="tRNA-Trp"

/product="tRNA-Trp"

/note="trnW-(tca)"

/anticodon=(pos:11611..11613,aa:Trp,seq:tca)

/gene="tRNA-Trp"

gene complement(11639..11701)

/gene="tRNA-Cys"

tRNA complement(11639..11701)

/gene="tRNA-Cys"

/product="tRNA-Cys"

/note="trnC (gca)"

/anticodon=(pos:complement(11670..11672),aa:Cys,seq:gca)

/gene="tRNA-Cys"

gene complement(11706..11772)

/gene="tRNA-Tyr"

tRNA complement(11706..11772)

/gene="tRNA-Tyr"

/product="tRNA-Tyr"

/note="trnY (gta)"

/anticodon=(pos:complement(11739..11741),aa:Tyr,seq:gta)

/gene="tRNA-Tyr"

gene 11775..13316

/gene="COI"

/note="Start codon ATT/ final codon TAA"

CDS 11775..13316

/gene="COI"

/codon_start=1

/transl_table=5

/product="Cytochrome c oxidase subunit I"

/translation="MMKKWLFSTNHKDIGTMYFILGIWSGLLGTTLSMMIRMELGNPG

SLLANDQLYNTIVTAHAFIMIFFMVMPIMIGGFGNWLVPLMLGAPDMAFPRMNNMSFW

LLPPSLMLLISSSIVESGSGTGWTVYPPLSSNIAHSGASVDLTIFSLHLAGISSILGA

INFITTIINMRSDSLSLDQMPLFVWAVGITAILLLLSLPVLAGAITMLLTDRNLNTSF

FDPAGGGDPILYQHLFWFFGHPEVYILILPGFGIISHIIAEESGKKETFGYLGMVYAM

MAIGFLGFIVWAHHMFTVGMDIDTRAYFTAATMIIAVPTGIKVFSWLATMYGSNINFS

PSMLWSLGFLFLFTVGGLTGIILANSSIDVSLHDTYYVVAHFHYVLSMGAVFAIMGGF

VHWYTLFTGLKLDNFKLKIQFFMMFTGVNITFFPQHFLGLSGMPRRYSDYPDCYTSWN

IVSSMGSFISLMSLMFLIFITWNSMINPQYALLNSNLSYSIEWNQTYPPYEHSYNELP

LLSNF*"

gene 13312..13380

/gene="tRNA-Leu"

tRNA 13312..13380

/gene="tRNA-Leu"

/product="tRNA-Leu"

/note="trnL2(taa)"

/anticodon=(pos:13342..13344,aa:Leu,seq:taa)

/gene="tRNA-Leu"

gene 13381..14094

/gene="COII"

/note="Start codon ATG/ final codon TAA"

CDS 13381..14094

/gene="COII"

/codon_start=1

/transl_table=5

/product="Cytochrome c oxidase subunit II"

/translation="MATWSNLKLQDSASPIMEQIDFIHDHALIILSMITVLVGYIMSY

ILINKFTNRYILDNQLIELIWTVIPAITLIFIAFPSLRLLYLMEEINNPFLTIKSIGN

QWYWSYEYSDFNNIEFDSYMIPTNDLSENSFRLLDVDNRIVLPMNNHIRFMVTASDVL

HSWTIPAIGVKMDANPGRLNQTSFFLKRPGIFYGQCSEICGANHSFMPIVMESIPTMS

FINWIKKNSLDDWKQVLVS*"

gene 14060..14130

/gene="tRNA-Lys"

tRNA 14060..14130

/gene="tRNA-Lys"

/product="tRNA-Lys"

/note="trnK (ctt)"

/anticodon=(pos:14090..14092,aa:Lys,seq:ctt)

/gene="tRNA-Lys"

gene 14130..14197

/gene="tRNA-Asp"

tRNA 14130..14197

/gene="tRNA-Asp"

/product="tRNA-Asp"

/note="trnD (gtc)"

/anticodon=(pos:14162..14164,aa:Asp,seq:gtc)

/gene="tRNA-Asp"

gene 14198..14359

/gene="ATP8"

/note="Start codon ATT/ final codon TAA"

CDS 14198..14359

/gene="ATP8"

/codon_start=1

/transl_table=5

/product="ATP synthase F0 subunit 8"

/translation="MPQMMPINWIFLFLMFITIFLLFIIMNYFNYNIKIEKKNYLKNN

NFKNLNWKW*"

gene 14353..15030

/gene="ATP6"

/note="Start codon ATG/ final codon TAA"

CDS 14353..15030

/gene="ATP6"

/codon_start=1

/transl_table=5

/product="ATP synthase F0 subunit 6"

/translation="MMTNLFSIFDPSTNLFFFSLNWLSALLGILFFPYIFWFLPNRYS

IFWNMINMKLHNEFKTLLNYENNKGSTFIFISLFLFILFNNFLGLFPYIFTSTSHLTL

TLSISLPLWISFMLYGWIINTQHMFTHLIPQGTPFILMPFMVLIETISNIIRPGTLAV

RLTANMIAGHLLLTLLSNNGCKISNLLVTFLILVQIILLVLESAVAVIQSYVIAILSM

LYSSEVN*"

gene 15039..15827

/gene="COIII"

/note="Start codon ATG/ final codon TAA"

CDS 15039..15827

/gene="COIII"

/codon_start=1

/transl_table=5

/product="Cytochrome c oxidase subunit III"

/translation="MSNFNNHPFHLVDYSPWPLTGAIGTLTLTAGMVKWFHNFNFFMV

LIGYTIVLLTMYQWWRDICREGTYQGKHTNLVAKGLRWGMILFIVSEVFFFISFFWAF

FHSSLAPNIEIGMSWPPMNIVPFNPFQIPLLNTIILISSGISITWAHHAIMDNNYTQT

LQGLLLTIILGIYFTILQAYEYNQAPFTISDSIYGSTFFMATGFHGLHVIIGTIFLAI

CFLRHLNNHFSSTHHFGFEAATWYWHFVDVVWLFLYISIYWWGF*"

gene 15830..15896

/gene="tRNA-Gly"

tRNA 15830..15896

/gene="tRNA-Gly"

/product="tRNA-Gly"

/note="trnG (tcc)"

/anticodon=(pos:15860..15862,aa:Gly,seq:tcc)

/gene="tRNA-Gly"

gene 15897..16250

/gene="NADH3"

/note="Start codon ATG/ final codon TAA"

CDS 15897..16250

/gene="NADH3"

/codon_start=1

/transl_table=5

/product="NADH dehydrogenase subunit 3"

/translation="MFFMNFMTLFFFIISLIMITLSYVLSKKSNNDREKCSPFECGFN

PKSSARVPFSLHFFLITVIFLIFDIEIALILPMIPLFNLVNMYMWSKITIFFLIVLLL

GVYHEWNQGMLNWTN*"

gene 16249..16313

/gene="tRNA-Ala"

tRNA 16249..16313

/gene="tRNA-Ala"

/product="tRNA-Ala"

/note="trnA (tgc)"

/anticodon=(pos:16279..16281,aa:Ala,seq:tgc)

/gene="tRNA-Ala"

gene 16314..16376

/gene="tRNA-Arg"

tRNA 16314..16376

/gene="tRNA-Arg"

/product="tRNA-Arg"

/note="trnR (tcg)"

/anticodon=(pos:16342..16344,aa:Arg,seq:tcg)

/gene="tRNA-Arg"

BASE COUNT 6799 a 1731 c 1238 g 6639 t

ORIGIN

1 cactgttaat gataacattg aattttaaaa ttccaattaa agaaatattt taaaattaag

61 ctgctaactt aattaaagtg gttaaaatcc attaatattt ctaataattt atatagttta

121 acaaaaacat tacattttca ttgtaaaatt aaataaattt attttataaa taaaatatat

181 ttaaaaatta aaataatttc cctgatatct tcaatatcat actctaatat ataagctatt

241 taaattaaat aataataata ttaaatatta tattaaataa taattataat ataaatcatt

301 attcataaaa caaatctaaa taaataaatt tttaaattat ttaattgaaa aaaataataa

361 aaaatagaat agaattttaa agtattaaac aatccttgac ctcttaaata ttcaactcaa

421 ccccgatcat ttttatataa aatttgacca aattttaaaa aattataatt aatacaatag

481 gttcttaatc caactataaa tcatatagtg gaaaaaaaaa atctaatttt ataagttaat

541 ataaatttat ttaaaaaatt ataattaaat aaactaacca agtaacctaa taatattccc

601 actaatctaa cataaataac tataattttt atatgtaaaa atatataaat aatatttaca

661 tcataaaata ataatcaaga taatattctc cctctaatta aacttataaa taaaagaaca

721 aatatactat aaattatatt ataatcttct tcaaataaat tataaattga aactaaatta

781 aaatctctaa ttattaaata aaataatatt cgaaatgtat aaaaaacagt taaacctgta

841 gataaataaa aaataacata aataaaaata ttaatattta ttatagaaat tctatctaac

901 attaaatcct tagaataaaa acccgatata aaaggtaaac cacataaaga taaattagaa

961 attactatac ataaagaagt taagggaata tacttacata taccccctat ataacgaata

1021 tcttgtatat caaatattat atgaataatt aaacctgcac atataaataa taaagcttta

1081 aatatagcat gagttaataa atgaaaaaaa gctaatttag gaaatcctat acataaaact

1141 cttattatta aacctaactg acttaaagta gaaaaagcaa taattttttt taaatcaaat

1201 tcataattag caacaattcc agctataaat attgttaaag tagataataa taataaaaat

1261 tttaaaacat atattcctat taataaatct ctaaaacgaa ttaatagata aaccccagct

1321 gttactaaag tagaagaatg aactaaagca gaaacaggtg taggagctgc tattgcagca

1381 ggaagtcaag aactaaaagg aatttgagca cttttagtta tagctgcaat tacaattata

1441 aaactaatta tttgtataga aaaatcattt tttataaagt ttaaataaaa aatataattt

1501 catctaccat aatttaatat tcaagaaata caaattaaaa taaaaacatc accaattcga

1561 ttagaaagag cagttaatat cccagaatta tatgatttat aactttgata ataaattact

1621 aaacaataag aaattaaacc taatccatct caacctaata aaattctaat taaatttggt

1681 ctaataatta ataatattat agaaaataca aataataaaa ctaataaaat aaaacgaact

1741 aaattcttat ctgattctat atatctattt ctatatttaa taactactga agaaattaat

1801 aaaacaaatc ttataaataa taaagatatt caatcaatta aaatagatat ataaacaaca

1861 tttgaattaa aagaaaatac ttcccattca ataaataaaa ctaaattctt aattatgaaa

1921 taaataaaaa aaaagaaatt tattaatcta aaaaaaaaaa gaaaaaaaaa tctattaaaa

1981 caaataaaat ttttattaat tatctaaagt aatgtttata ttacatattt gactccacaa

2041 atcaatattt taattaaatt atttaaataa ttaaaattgt tatttatata atttaattaa

2101 attcaaatta aaaaataatc aaccttaaga attaaaagat ttaaaggaag tcaatgtaat

2161 aataataata aatactctcg agaagtacct ctataaaatc tataaattcc tttgaaaatt

2221 atcccatgtt gagtatatga atataaatat aatctataag cagctctaaa aaaagaaact

2281 aatattaata aaaatattct taatcaagat caagataata atctattgat taaactaatt

2341 tccccagcta aatttaaaga tggtggacaa gatatattcg aagataataa aaggaatcat

2401 catattctta tagaaggtat aaaatttatt atacccttat taactaataa tcttcgtctt

2461 cttaagcgtt cataatttat attagataaa caaaacattc ctgaagaaca taatccatga

2521 ccaattatta taatataaga tcccataaat cctcaataat ttatagttat aataccccca

2581 attactaatc ttatatgagc tacagaagaa tatgcaatta aagccttaat atcaatttga

2641 actaaacaga ctaatctaat ataaacacca ccaattaatc taataataat tcaaataaaa

2701 cttaatttta cagaaatttc ttgtaaaaaa atcattactc gtattaatcc ataacctcct

2761 aattttaata taatagcagc taaaattatt gaacctgaaa taggagcttc tacatgagcc

2821 ttaggtaatc ataaatgaac aaaatatata ggcattttaa ctaaaaaagc taaaattata

2881 acaatataaa gataaaaaaa agtaaaatta taaaatttta atatataaaa tcttaaacaa

2941 tttaaatttt tgtataaaaa aaaaattcct attaatatag gtaaagaggc aaataaagta

3001 taaaataata aataaagtcc tgcttgtata cgttcaggtt gataacccca tcctacaatt

3061 aatattaaag taggaattaa tctaccttca aaaaaaaaat aaaatataaa taaatttatt

3121 gttgagaaag ttaatattaa tataactatt aaaataatta aattaattaa aaatccatta

3181 agaaaaaaat ttaatttata taaagactca cttgaagtaa ttattaaaca acaaactcaa

3241 attcttagta aaattaatcc ataagatatt atatcacaac caaatccata tctaatcgaa

3301 tatatacccc ctaaactaat ataatttaat aaaaataaaa aaaataataa aaatattatt

3361 atttgaacca ttcaaaatat attaaaaaaa aaacataaag gtattataaa taataaaaaa

3421 agaaaaaatt ttatcattaa ttaattaatt taataaattg aaactttgaa aataatcatt

3481 tccatgacta cgaattattg atactaatac agataaacct aataccccct cacaaactga

3541 aaatactaaa aaaactatta atatatatat atcacaatta taatatatta aaaatactaa

3601 taaaaaaata aaaattctta aaactaaata ttctaaactt aataaaacaa ttattaaatg

3661 tttatattta gaaacaaaaa ttatattacc aataaaaaat ataaaaaaaa ttatttttat

3721 catttgtaaa ataaaaaaat tttagttttt atagtttaag aaaaacattg gtcttgtaaa

3781 ccaaaattaa aaaatttttt ttaaaaactt caaagaaaaa gattttcttt atcaataatc

3841 tccaaaatta ttattttatt taaactattc tttgaaatta aaattatttt aataggtcta

3901 attattaatt tatcattttt aatatatttt ctaaaccatc cattaactat aggatttata

3961 attatcgtac aaaccttatt aacatgttta atatctgggt tattattaaa tacttattga

4021 ttttcttata ttttattttt aaccttttta gggggaatat tagtattatt tatttatgtt

4081 tcaagaattg catcaaatga attattttat aactcaaaat atttattaat aatattaatt

4141 ataaattttt tcttattatt tttaatatat attatttttt ataataagga aatagaaatt

4201 aaaaatatta ataatgaaat aaataaatta aactatgaat tttttatatt taattatgaa

4261 tataaaatta atatttctaa aatatataat aatcaatcac catttataat atatatatta

4321 attatttatt tatttattac attaattgca gtagtaaaaa ttaataatat tttttatgga

4381 cccttacgtt catcaaactt ctaaccaatg aaaaataatt tcaaatctat tttaaaaact

4441 cacccaattt taaaaattat taatggatca ttaattaatt taccttcccc atctaatatt

4501 tcaatttgat gaaattttgg ttctctttta ggattatgtt taataattca aattattaca

4561 ggattatttt taactatata ttataccgca aatattgata tagcattttt tagagttaat

4621 tatatttgcc gaaatgtaaa ttatggatga ttaatccgaa ctatgcatgc caatggagca

4681 tctttatttt ttatttgtat tttccttcat attggccgag gaatatatta tgaatctttt

4741 attttaaaag taacctgatt agtcggaact ttaattttat ttattttaat agcaacagca

4801 tttttaggat atgttttacc ttgaggtcaa atatcatttt gaggggcaac agtaattact

4861 aatttattat cggctattcc ttatttagga aatactcttg tagtatgaat ttgaggagga

4921 tttgcaattg ataatgcaac attaacgcga ttttatacat tccattttat cttacccttt

4981 gttattttaa taatatctat aatccattta atatttcttc atcaaacagg atcaaataat

5041 ccattaggaa ttaaaagaaa tatagataaa atcccattcc atccattttt tacttgaaag

5101 gatattttag gatttattgt agtaattttt ttattcttaa tattagtttt aattaaccct

5161 aattatttag gagatccaga taattttatt cctgctaatc cattagtaac tcctgctcat

5221 attaaaccag aatgatattt cttatttgct tatgcaattt tacgatcaat tcctaacaaa

5281 ttaggaggag taattgcttt aattttttca attttaattt taataacttt accttttact

5341 catttaaaaa aaactcaagg tatccaattt tatccattaa atcaaattta tttttgatct

5401 attgtagtta cattaatttt attaacatga attggagctc gacctgttga agatccatat

5461 attacaacag gacaaatttt agctatttat tatttttcat actttatttt taatcctctt

5521 ctaggaaaat tatgagatga ttatttattt aaatataatt aattaattaa tgagcttgta

5581 aaagcatttg ttttgaaaac ttaagaaaga atttttattc tattaattta aactaaatta

5641 aattattaaa taaatataat ttttaatcca acaaaaaaaa gtaaaaaatt taaagaaact

5701 ggtaaataaa tttttcaagc taaatatatt aatttatcat aacgataacg aggtaaagtt

5761 cctcgaactc aaacaaataa aaatgaaata aaagttaatt taaaataaaa taaatataaa

5821 gaataacctc ctatatataa taatacaaat aatattctta taaataaaat tcttgaatat

5881 tcagctaaaa aaattaatgc aaatccccct cttctatatt caatattaaa tccagaaact

5941 aattctcttt ccccctctgc aaaatcaaag ggagttcgat ttgtttcagc taatatagat

6001 gaaattatta ttaatcttaa aggaattata taaattataa atcatataaa cttttgataa

6061 attataaaat taaataaacc aaaatccatt aataaaataa ttctagatat aataattaaa

6121 actaatctaa cttcataaga aatagtttga gctacagaac gtaatccccc taataaagca

6181 taattagaat tagaagacca accagctaat ataactgtat aaactcctat tcttgttacg

6241 caaaaaaaaa ataatatccc taaattaaat ctaactaaat taaaataata aggaattaat

6301 aatcaaataa ttaaagaaaa taaaaatcta aatactggag aaaaataata aacaataaaa

6361 tttgaatata ttggataagt ttgctcctta gtaaataatt taattccatc agaaaaaggc

6421 tgtaaaatcc ctattaatcc taccttatta ggtcctttac gaaattgaat ataacctaat

6481 aatttacgtt ctaataaagt taaaaaagcc accccaatta aaaccccaat aaataatact

6541 aataagctaa aaaaaattat taaattatcc ttaattatta aaattactac ttatataata

6601 taaaaattat atataaatga tttctaaggc catcacatct ttctgccaaa gtagtcaaat

6661 aaattaataa aattcaattt tataaaatta tttttaatat taattccttt cgtactaaaa

6721 tattaatatt atctaaagat agaaaccaac ctggctcaca ccggtttgaa ctcagatcat

6781 gtaagatttt aatgatcgaa cagatcaaaa ttttaaactt ctgcatttaa attttatctt

6841 aatccaacat cgaggtcgca aactcttttt tttattcgaa ctaaaaaaaa aaattacgct

6901 gttatcccta aggtaattta atcttttaat ctttaataaa aaatcattat ttcataaata

6961 aatgtaaaaa agaaaaaaaa gtttattaaa tttttttatc accccaatta aattaattat

7021 taaatttaaa tttaaaatta tataattaat ataaataaaa taattaatta aactctatag

7081 ggtcttctcg tcttttaaaa atattttaac tttttaaata aaaaattaat ttctattaat

7141 ttatattgag acagcttata tttcatccaa tcattcattc tagtcaccaa ttaagagact

7201 aatgattatg ctacctttgt acagtcaata tactgcagcc cttcaatttt tataaatcag

7261 tgggcagatt agactttaaa ttattttcaa aaagacatgt ttttgataaa caggtgaata

7321 ttaattttgc cgaattcctt aaaataaatt ttttttttaa aaaaaattta atataaatat

7381 attatatact aattttatca ttatatctta atttatatta ttataattaa tttttttcta

7441 aaaaattaaa ttaaatttaa attttatttt tattaaaatt ttttataaaa aactaaaatt

7501 tattaaaaat ttaatttata taaattttaa taataaaatt tttattaatt ataaaaattt

7561 aatttaaagc ttatccctta aaatatttaa ttttttcata atttattttt ataatataaa

7621 taaattaatt ataaaaaatt ataaattaaa tttatttcaa aaaaaactag atatcttaaa

7681 aaacgtataa catttcattt ccaattaatt attaaaaata tttatgcaac aataaatttt

7741 ttaattaatt ttctcttttt aattcgagaa tattatataa attaatattt aattaataaa

7801 ctctgataca caagatacat taaaataaaa ttacttttta aaaattttat tttcaactaa

7861 tatttcaatt ttctttcaca atactattta tctataaata aattaatttt ttcttttaat

7921 aatactttaa cccccctatt aaatgattat taaaattaaa attattttta ttaatttttt

7981 attattaaat tttttattat caagttaagt gaattttatc actataattt caatgtaaat

8041 gaaatacttt attaagatat aacttgtctt tctagaaaca ctttccagta cctctacttt

8101 gttacgactt attctaattt ataaatagaa gtgacgggca atatgtacat attttaattt

8161 aaaatcattt tattataata aataaaatta catttaaatc caattttaat taaattttcc

8221 aatttaatat ccatttaaat aaatttattg taatccatta tattcttaat tataagctac

8281 atcatgattt gatttaattt catattaaaa ttctaaaata ttataatttt taaaaaatat

8341 ttttttaaca acgatatata aactataaaa attaagtaaa tttaatcgtg gattatcaat

8401 taataaacag attcctctaa ataaaataaa ataccgccaa cttaattaag tttctttaaa

8461 taattaaata ctattttagt atattaattt aagtttttat aatagggtat ctaatcctag

8521 ttttttaaaa aatttattaa attatcattt attaaaatta aattttttta aaattaaaat

8581 ttcacctaat aattttaaat ttaatttaaa tttatttata taaattaatt aattactaat

8641 aaaatttaat ttatcttttg tttaaccgca actgctggca caaaatttgt taataatctt

8701 tatattacta aatcataatt ttttatataa tttaatttta attactacaa ataaaaataa

8761 ttattattaa aataatcatt tataactaaa atttatatgt aaaatatatt tatattaaat

8821 ttttttaact ttaaaaatta tttatttaat tacataattt tttatataga tttttttttt

8881 ttttttttta tattaatata tttatatata attatatatt taatataata tattaaaatt

8941 atttaaattt atattaaaat aattaataat aattattaaa tttttaatat tttctcccta

9001 tttttttaat aatattaata ttaaaaatta atataattaa taaacctata tagttaattt

9061 atataactat atattaatat atttatataa attataggtt aaatttaata tattaatata

9121 tttatatata attatatatt taatataata tattaaaatt atttaaattt atattaaaat

9181 aattaataat aattattaaa tttttaatat tttctcccta tttttttaat aatattaata

9241 ttaaaaatta atataattaa taaacctata tagttaattt atataactat atattaatat

9301 atttatataa attataggtt aaatttaata tattaatata tttatatata attatatatt

9361 taatataata tattaaaatt atttaaattt atattaaaat aattaataat aattattaaa

9421 tttttaatat tttctcccta tttttttaat aatattaata ttaaaaatta atataattaa

9481 taaacctata tagttaattt atataactat atattaatat atttatataa attataggtt

9541 aaatttaata tattaatata tttatatata attatatatt taatataata tattaaaatt

9601 atttaaattt atattaaaat aattaataat aattattaaa tttttaatat tttctcccta

9661 tttttttaat aatattaata ttaaaaatta atataattaa taaacctata tagttaattt

9721 atataactat atattaatat atttatataa attataggtt aaatttaata tattaatata

9781 tttatatata attatatatt taatataata tattaaaatt atttaaattt atattaaaat

9841 aattaataat aattattaaa tttttaatat tttctcccta tttttttaat aatattaata

9901 ttaaaaatta atataattaa taaacctata tagttaattt atataactat atattaatat

9961 atttatataa attataggtt aaatttaata tattaatata tttatatata attatatatt

10021 taatataata tattaaaatt atttaaattt atattaaaat aattaataat aattattaaa

10081 tttttaatat tttctcccta tttttttaat aatattaata ttaaaaatta atataattaa

10141 taaacctata tagttaattt atataactat atattaatat atttatataa attataggtt

10201 aaatttaata tattaatata tttatatata attatatatt taatataata tattaaaatt

10261 ttaagttaga ccattcataa taaatattta tattaatata attttttaaa tttaaaaata

10321 agctaaatta agcttttggg ttcatacccc aactataaag gaaatccctt ttttttaaaa

10381 aaaataaagt gcctgatgaa aaggattact ctgatagggt aaataatgta attaaaatta

10441 cctttattat attttataga attaaactat acccaataga atcaaaatct attatgcatc

10501 ttacactaaa atatatttta ttaaatttaa aatttaatat ttttattctt aaataaatat

10561 tttatattaa ttatattttt attaattcaa ataaaatatt ttttctattt attttaataa

10621 ttagaaccat aatctcaatt tcttcaaatt cttggtttgg atgttgaatt ggattagaaa

10681 ttaatctact tagatttatc ccccttattt ctaatagaaa aaatctttta tcctcagaag

10741 catcattaaa atattttctt actcaatcaa ttgcgtcaat taatttatta tttttaatta

10801 ttatgataag attttttaaa caattagaaa attataatat tataatatta ttaataaatt

10861 tacctttatt aataaaaata ggatcagcac cctttcattt ttgatttcct aatttaattg

10921 aaggattatc ttgaataaat gcttatatta ttataacatg acaaaaaatt acccccttaa

10981 ttttaatctc ttataattat aacttttatt taattagatt tattgctatt ttaaatgcct

11041 ttgttggagc tattggaggt tttaatcaaa tatctttacg aaaaatttta actttctctt

11101 ctattaataa tttaagatga atattaattt caattataat tagagaaaat ttatgaatat

11161 tatatttttt tatttattgt attttaaatt ttatattatg ttattttttt aatttaataa

11221 atatttttta tattaatcaa atttattttt ataatataaa aaatttttat aaaattttac

11281 ttgcaataaa tttattttct ttaggtggat taccaccatt tatagggttc ttatcaaaat

11341 gattagttat taatttttta attaataata acatattaat attaactttt ttaataatca

11401 tatcaagttt aattatatta tactattata ttcgaattat atattcaact tttttaatta

11461 attatataaa attaaaatga ttcaatttca aaattaaaaa taattttaat aaatattatt

11521 ttatttgtat atatatattt agatttttag gaattcctat tagaacttta accttttttt

11581 aaggttttaa gttatttaaa ctaataatct tcaaaattat aaaaaaagaa ctttctttaa

11641 gccttagaat ttttattcac cttaaaattt gcaattttat atcataaatg aatataaggc

11701 ttaaataata aaaaagaatt aaatcttgtt aataaattta caatttatcg cttaaacctc

11761 agccatttta ttttattata aaaaaatgat tattctcaac aaatcataaa gacattggaa

11821 ctatatattt cattttagga atttgatcag gattattagg tacaacttta agtataataa

11881 ttcgaataga attaggaaat cctggatcac ttttagcaaa tgatcaactt tataatacta

11941 ttgttacagc acatgctttc attataattt tctttatagt tatacctatt ataattggag

12001 gatttggaaa ttgattagta cctttaatat taggagcacc agatatagct tttcctcgaa

12061 taaataatat aagattttga ttattacctc catctttaat acttttaatt tctagaagaa

12121 tcgtagaaag tggatcagga actggatgaa ctgtttatcc tcctctttca tctaatattg

12181 cccatagagg agcttctgta gatttaacaa ttttctctct tcatttagct ggaatttcat

12241 caattttagg agcaattaat tttattacaa ctattattaa tatacgaagt gatagattat

12301 ctcttgatca aataccttta tttgtttgag cagtaggaat tacagcaatt cttttattat

12361 tatcattacc agttttagct ggagctatta caatattatt aactgatcga aatttaaata

12421 cttcattttt tgatccagct ggaggaggag atccaattct ttatcaacat ttattttgat

12481 tttttggaca tcctgaagta tatattttaa ttttaccagg atttggaatt atttctcata

12541 ttattgctga agaaagagga aaaaaggaaa cttttggata cttaggaata gtatatgcaa

12601 taatagcaat tggattttta ggatttattg tatgagctca tcatatattt accgtaggta

12661 tagatattga tacccgagca tactttacag cagctacaat aattattgct gtacccactg

12721 gtattaaggt atttagttga ttagctacta tatacggatc taatattaat ttttcacctt

12781 ctatattatg aagattagga tttttattct tatttacggt aggaggatta acaggaatta

12841 ttttagctaa ttcatctatt gatgtatctt tacatgatac atattatgta gtagcacatt

12901 tccattatgt tttatctata ggagctgtat ttgcaattat aggaggattt gttcattgat

12961 atactttatt tacaggttta aaattagata attttaaatt aaaaattcaa ttctttataa

13021 tgtttacagg ggtaaatatt acatttttcc ctcaacattt tttaggatta tctggaatac

13081 ctcgtcgata ttcagattat ccagattgtt atacttcatg aaatattgta tcatctatag

13141 gatcatttat ttctttaata agattaatat ttttaatttt tattacatga aattcaataa

13201 ttaaccctca atatgcttta ctaaactcta atttaagtta ttcaattgaa tgaaatcaaa

13261 cttatccacc atatgaacat tcatataatg aattaccatt actaagaaat ttctaatatg

13321 gcagattaat tgtaatggat ttaaacccca tttataaagg attaaatcct ttttttagaa

13381 atggcaactt gatcaaacct aaaactacaa gatagagctt ccccaattat agaacaaatt

13441 gattttattc atgatcatgc tttaattatt ttatctataa ttactgtttt agtaggatat

13501 attatatctt atattttaat taataagttt acaaatcgtt atattttaga taatcaatta

13561 attgaattaa tttgaactgt aatcccagct attacattaa tttttattgc tttcccatct

13621 cttcgattat tatatttaat ggaagaaatt aataatccat ttctaactat taaatctatt

13681 ggaaatcaat gatattgaag ttatgaatat tcagatttta ataatattga gtttgattct

13741 tatataattc caacaaatga tttaagtgaa aatagttttc gattattaga tgtggataat

13801 cgaattgttt taccaataaa taatcatatt cgatttatag taactgcaag agatgttctt

13861 cattcttgaa ctattccagc tattggagta aaaatagacg caaatccagg tcgactaaat

13921 caaacaagtt tttttttaaa acgaccaggt attttttatg gacaatgttc agaaatttgt

13981 ggagcaaatc acagttttat acctattgtt atagaaagta ttcccactat aagatttatt

14041 aattgaatca aaaaaaactc attagatgac tgaaagcaag tactggtctc ttaaaccatt

14101 ttatagtaat ttagcaatta cttctaatga aagaattagt taaaatatat aacataaata

14161 tgtcaaattt aaattattat aaatataata ttcttttatt ccacaaataa tacctattaa

14221 ttgaattttt ttatttttaa tatttattac tattttctta ttatttatta ttataaatta

14281 ttttaattac aatattaaaa ttgaaaaaaa aaattattta aaaaataata attttaaaaa

14341 tttaaattga aaatgataac aaatttattt tccatttttg atccctcaac taatttattt

14401 tttttctcat taaattgatt aagagcttta ttaggaattt tattttttcc ttatattttt

14461 tgatttttac ctaatcgata ttcaattttt tgaaatataa ttaatataaa attacataat

14521 gaatttaaaa ctttattaaa ttatgaaaat aataaaggat caacatttat ttttatttca

14581 ttatttttat ttattttatt taataatttt ttaggattat ttccttacat ttttacaagt

14641 actagtcatt taaccttaac attatcaatt tcattacctt tatgaattag atttatatta

14701 tacggatgaa ttattaatac acaacatata tttacccacc ttattcccca aggaacacct

14761 tttattttaa taccatttat agtattaatt gaaactatta gaaatattat tcgacctgga

14821 actttagctg tacgattaac tgctaatata attgcaggac atttattatt aactttatta

14881 agtaataatg gatgtaaaat ttctaattta ttagtaactt ttttaatttt agtacaaatt

14941 atcttattag tattagaatc agcagttgca gttattcaat cttatgtaat tgccatttta

15001 agtatacttt attctagaga agtaaattaa tttaaccaat gtcaaatttt aataatcatc

15061 cattccattt agtagattat agaccatgac ctttaacagg tgcaattgga acattaactt

15121 taactgctgg aatagttaaa tgattccata attttaattt ttttatagtt ttaatcggat

15181 atacaattgt tttattaact atatatcaat gatgacgaga tatttgccga gaaggaactt

15241 atcaaggtaa acatacaaat ttagtagcaa agggattacg atgaggaata attttattta

15301 ttgtatcaga agtatttttt tttatttctt ttttttgagc ttttttccac agaagtttag

15361 cccctaatat tgaaattggt atatcatgac ccccaataaa tattgtacca tttaatccat

15421 tccaaattcc tttattaaat acaattattt taatttcatc aggtatttct attacatgag

15481 cccatcatgc aattatagat aataattaca ctcaaaccct tcaaggatta ttattaacaa

15541 ttattttagg aatttacttt actattttac aagcatatga atataatcaa gctcctttta

15601 ctatttcaga tagaatttat ggatcaacat tttttatagc aacaggattc catggtcttc

15661 atgtaattat tggaactatc ttcttagcaa tttgtttctt acgacattta aataatcact

15721 tttctagaac tcatcatttt ggatttgaag ctgcaacttg atattgacat tttgttgatg

15781 tagtatgatt attcttatat attagaattt attgatgagg attttaatta tttatataat

15841 atatttagta tatttgattt ccaatcaaaa agtttaatta taatttaata taaataattt

15901 tttttataaa tttcataaca ttattttttt ttattatctc cttaattata attactttat

15961 cttatgtatt atcaaaaaaa tctaataatg atcgagaaaa atgctcacca tttgaatgtg

16021 gatttaaccc taaatcatca gcacgagtac cattttctct tcatttcttt ttaattacag

16081 taattttttt aatttttgat attgaaattg cattaatttt accaataatc cctcttttta

16141 atttagtaaa tatatatata tgatctaaaa ttactatctt ttttttaatt gttcttttat

16201 taggagttta tcatgaatga aatcaaggaa tattaaattg aacaaattag gattatagtt

16261 taaataaaac atttgatttg cattcaaaaa atattgaatt tcaatttatc ttaaaataag

16321 aagcaatttt tgcatttaat ttcgacttaa aagactgagt tattactcct tatttaatat

16381 ttaattgaaa ccaaaataga ggtatat

//
